# A new species from the genus *Lepechinelloides* Thurston, 1980 (Amphipoda, Lepechinellidae) from the Clarion-Clipperton Zone, Pacific Ocean

**DOI:** 10.3897/zookeys.1274.142630

**Published:** 2026-03-24

**Authors:** Rachael A. Peart, Anne-Nina Lörz

**Affiliations:** 1 New Zealand Institute of Earth Science Limited (Earth Sciences New Zealand), Wellington, New Zealand New Zealand Institute of Earth Science Limited (Earth Sciences New Zealand) Wellington New Zealand; 2 Marine Research at Schenckenberg am Meer, Südstrand 40, 26382, Wilhelmshaven, Germany Marine Research at Schenckenberg am Meer Wilhelmshaven Germany

**Keywords:** Abyssal, Clarion-Clipperton Zone, Crustacea, deep sea, Pacific Ocean

## Abstract

A new species of the genus *Lepechinelloides* is described from the Clarion-Clipperton Zone in the Pacific Ocean. *Lepechinelloides polymetallica***sp. nov**. is distinguished from the other two species in this genus by the ventrally directed midventral extensions on pleonites 1 to 3, widened propodi of both gnathopods, and small upright pointing extensions on all pereonites. This species is both morphologically and molecularly defined and compared to the other two species in the genus.

## Introduction

*Lepechinelloides* Thurston, 1980 is one of five genera within the family Lepechinellidae Schellenberg, 1926 and can be distinguished from the other genera based on the lack of a rostrum and cephalic teeth, possession of a non-triturative mandibular molar, a one-articulate mandibular palp, and the presence of peg-like protrusions on the head and body (Andres and Brandt 2001). There are only two species currently recorded from this genus, *Lepechinelloides karii* Thurston, 1980 and *Lepechinelloides weddellensis* Andres & Brandt, 2001. Both species occur in deep waters, *L. karii* from the East Icelandic Basin at 2450 m depth, and *L. weddellensis* from the Weddell Sea, Antarctica at 1983 m depth (Andres and Brandt 2001).

There is very little morphological variation within this genus, mainly focussed around the protrusions on the pereon and pleon. This study adds a distinct new species, *Lepechinelloides polymetallica* sp. nov. to this genus, based on a single specimen collected from the Clarion-Clipperton Zone at depths of 4223 m. We provide a description as well as molecular information of the new species.

## Methods

The material for the present study was sampled in the central-east Pacific Ocean, in the easternmost sector of the Clarion-Clipperton Zone (CCZ). The material studied was collected using an epibenthic sledge (EBS) during the expedition ABYSSLINE-2, ABYSSal baseLINE project (Smith et al. 2015). For details of sample processing see Jażdżewska and Horton (2026) and Jażdżewska et al. (2025).

The habitus of the holotype specimen is presented as a photograph obtained with a confocal laser scanning microscope (CLSM). The specimen was stained in Congo red and acid fuchsin, temporarily mounted onto slides with glycerol and examined with a Leica TCS SPV equipped with a Leica DM5000 B upright microscope and three visible-light lasers (DPSS 10 mW 561 nm; HeNe 10 mW 633 nm; Ar 100 mW 458, 476, 488 and 514 nm), combined with the software LAS AF 2.2.1 (Leica Application Suite, Advanced Fluorescence). A series of photographic stacks were obtained, collecting overlapping optical sections throughout the whole preparation (Michels and Büntzow 2010; Kamanli et al. 2017).

The specimen was then dissected and mounted on temporary slides using glycerol, and illustrations were made using a Nikon SMZ1500 microscope. All slides were examined using either a Nikon Eclipse Ci, or Zeiss compound microscope equipped with a camera lucida. Pencil drawings were scanned and manually inked and the plates constructed using Adobe Photoshop and a WACOM digitiser tablet. Slight differences are apparent in the illustrated coxae and CLSM owing to the three-dimensional status of this appendage. Appendages of the left side are dissected and illustrated, unless otherwise stated.

In the descriptions and figures the following abbreviations are used: A1, 2 = antenna 1, 2; c1–4 = coxa 1–4; G1, 2 = gnathopod 1, 2; LL = lower lip; Md = mandible; Mx1, 2 = maxilla 1, 2; Mxp = maxilliped; P3–7 = pereopod 3–7; U1–3 = uropod 1–3; UL = upper lip; T = telson; l = left; r = right.

Type material is deposited in the Senckenberg Museum in Frankfurt, Germany (SMF).

### DNA extraction, amplification, and sequencing

The holotype had its cytochrome *c* oxidase subunit I gene (*COI*) barcoded prior to identification of the species. The molecular procedures for samples collected on the ABYSSLINE-2 cruise are described in Jażdżewska et al. (2025).

## Results

### Systematics

**Order Amphipoda Latreille, 1816**

**Suborder Amphilochidea Boeck, 1871**

**Superfamily Dexaminoidea Leach, 1814**

**Family Lepechinellidae Schellenberg, 1926**

#### Genus Lepechinelloides

Taxon classificationAnimaliaAmphipodaLepechinellidae

Thurston, 1980

0FA63DCC-AAFB-5DA7-AED4-CCB3260EC29E

##### Diagnosis.

Body with large processes located along dorsal midline, never subdorsally; cuticular spines raised on peg-like projections. Urosome segments 2 and 3 coalesced. Head lacking eyes, rostrum, and cephalic teeth. Antenna 1 peduncle articles 2 and 3 subequal. Mandible: molar nontriturative; palp of one article. Lower lip inner lobes fleshy, well developed. Maxilla 1 palp biarticulate; maxilliped palp with four articles. Gnathopod 2 unguis of dactyl elongate. Peraeopods 3–4 carpus elongate. Uropod 1 rami immensely long (after Thurston 1980).

##### Type species.

*Lepechinelloides karii* Thurston, 1980.

##### Species composition.

*Lepechinelloides karii* Thurston, 1980; *Lepechinelloides polymetallica* sp. nov.; *Lepechinelloides weddellensis* Andres & Brandt, 2001.

#### Lepechinelloides polymetallica sp. nov.

Taxon classificationAnimaliaAmphipodaLepechinellidae

EC0B4789-9690-50C8-8321-485094D13D47

https://zoobank.org/0D9AC7D9-16D4-49A8-B256-215086CCAEBB

Figs 1, 2, 3, 4

##### Type material.

***Holotype***: Pacific • 3 mm; Clarion-Clipperton Zone; 12.045°N, 117.424°W, 4223 m; 16 March 2015; Ocean Mineral Singapore exploration contract area, RV *Thompson*, ABYSSLINE-2 Cruise, Station AB2-EB12; SMF 63356; COI (PQ734637).

**Figure 1.**
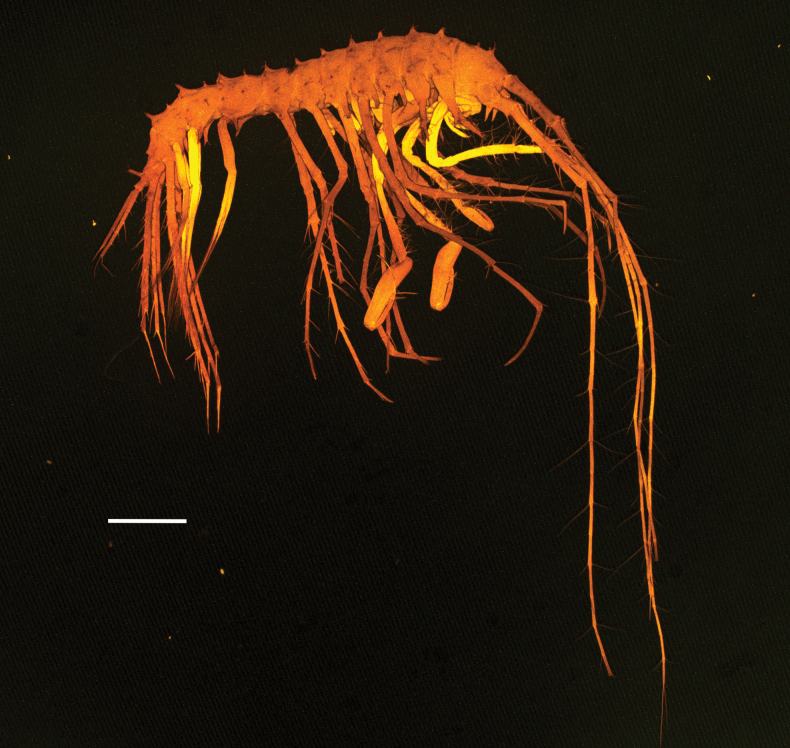
*Lepechinelloides polymetallica* sp. nov. Holotype, SMF 63356, 3 mm, CLSM scan. Scale bar: 0.5 mm.

**Figure 2.**
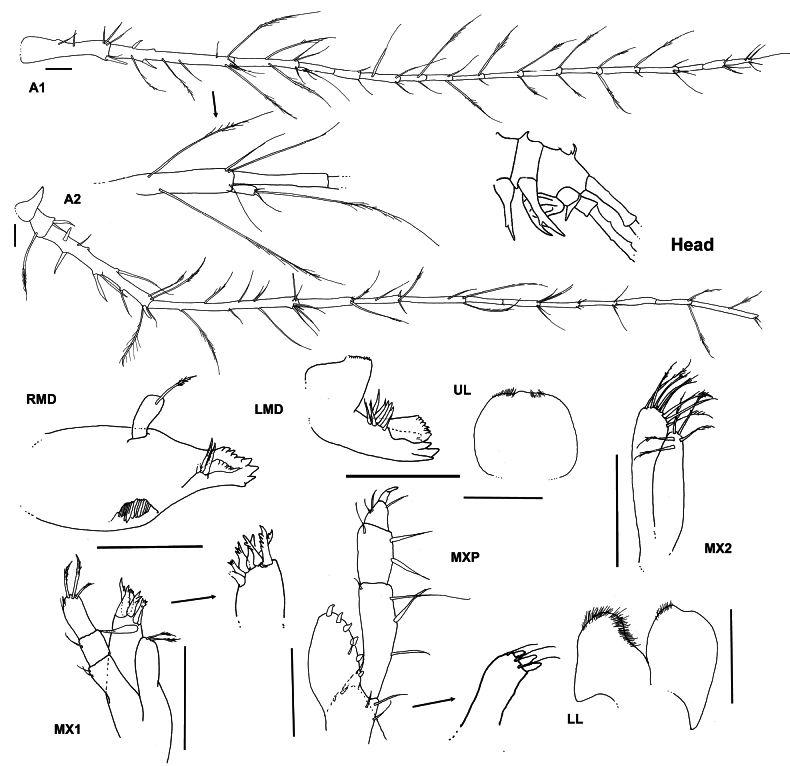
*Lepechinelloides polymetallica* sp. nov. Holotype, SMF 63356, 3 mm, A1 – 2, mouthparts. Scale bars: 0.1 mm.

**Figure 3.**
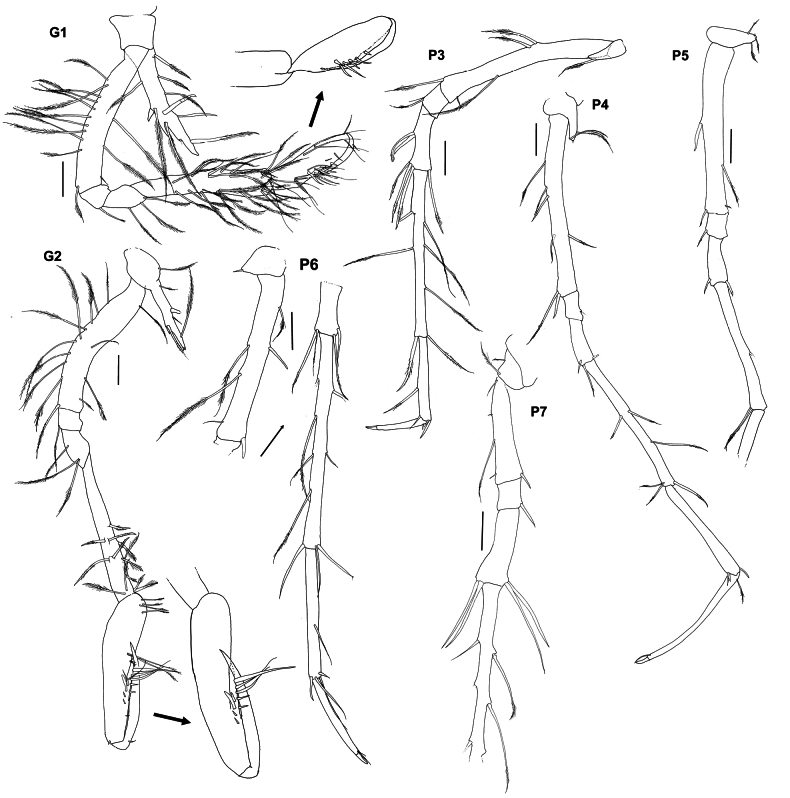
*Lepechinelloides polymetallica* sp. nov. Holotype, SMF 63356, 3 mm, G1 – 2, P3 – 7. Scale bars: 0.1 mm.

**Figure 4.**
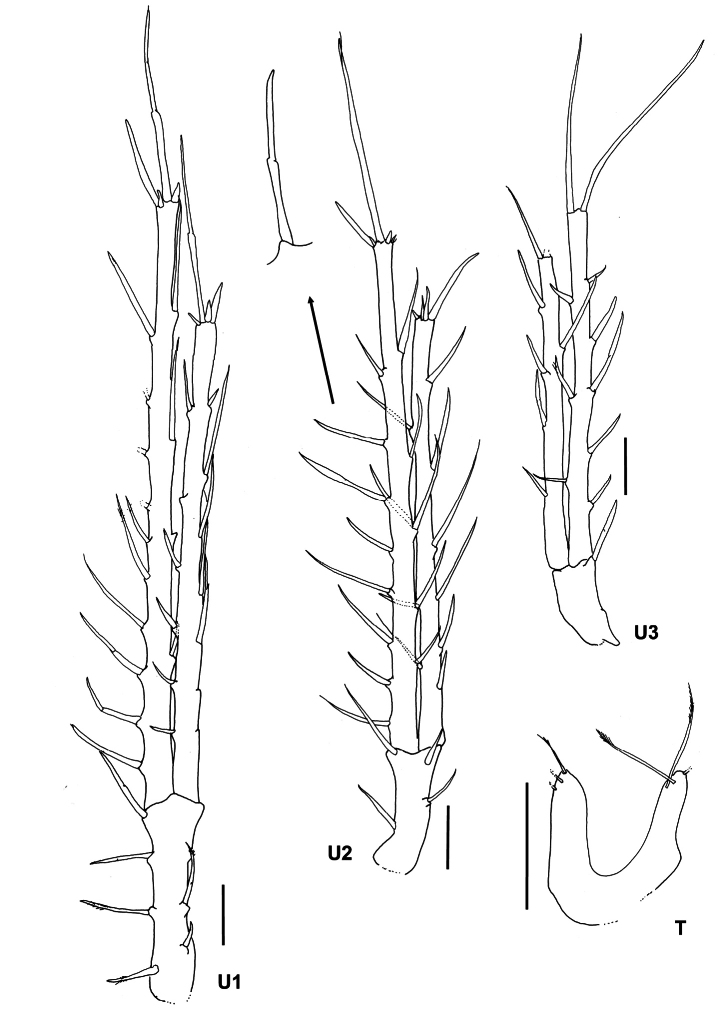
*Lepechinelloides polymetallica* sp. nov. Holotype, SMF 63356, 3 mm, U1 – 3, T, body lateral view. Scale bars: 0.1 mm.

##### Type locality.

Clarion-Clipperton Zone, 12.045°N, 117.424°W, 4223 m.

##### Etymology.

The species name polymetallica refers to the habitat where the new species was sampled, a polymetallic nodule field in the East Pacific; it is used as a noun in apposition.

##### Diagnosis.

Body not strongly setose, each pereonite with small upright projection dorsally. Head with two projections mediodorsally. Antenna 1 peduncle similar in length to the length of antenna 2 peduncle articles 4 and 5 combined. Accessory flagellum of one article, about one-third the length of the first article of flagellum, without aestethascs. Mandible right lacinia mobilis seven-dentate, left lacinia mobilis eight-dentate. Maxilla 2 apical margin of outer plate obliquely sloping medially. Maxilliped setation of palp articles 1–3 straight, not curved; palp article 4 unguiculate, unguis same length of basal part. Pereopod 3 without extra projections, without a spine apically. Pleonite 3 with small, dorsal, vertical directed projection.

##### Description.

Based on holotype, male, 3 mm, SMF 63356.

**Body** (Figs 1, 4) with sparse tuft-like projections (these are cuticular projections that have an apically or subapically inserted slender seta giving a “tufted” look) mainly along dorsal mid-line and a smaller in size lesser row mid-laterally, not strongly setose (see Fig. 1CLSM). ***Pereon*** (Figs 1, 4) all segments weakly laterally dentate with small dorsal tuft-like projections, pereonites 1–7 with main dorsal projection at the distal margin middorsally, all of similar size. ***Pleon*** (Figs 1, 4) each segment with one dominant and one smaller projection along the midline. Projections directed vertically and are relatively short. ***Epimera*** (Figs 1, 4) each with large acute tooth on the ventral margin, each directed ventrally; posterior margin reduced and angled. Ventral teeth smallest on epimeron 1, epimeron 2 and 3 of similar size. ***Urosome*** (Figs 1, 4) urosomite 1 weakly produced to form a small rounded distal dorsal tooth, and with rounded proximal hump; urosomites 2 and 3 fused, without projections.

**Head** (Figs 1, 4): two tufted dorsal projections; lacking rostrum and cephalic lobes. ***Antenna 1*** (Figs 1, 2) longer than body; first article of peduncle 0.7× length of second article, with sparse slender simple setae on margins; second article 2.0× length of third article, with one robust seta on each margin and sparse long slender plumose setae, third article 0.7× length of first article, with sparse long, slender, plumose setae; flagellum 1.6× peduncle, 14-articulate; accessory flagellum short, one articulate, reaching 0.3 × first article of flagellum. ***Antenna 2*** (Figs 1, 2) longer than antenna 1, peduncular article 4, 80% length of article 5; flagellum longer than peduncle, flagellum with 10 articles.

**Mouthparts** (Fig. 2): ***upper lip*** symmetrically rounded, almost quadrate, setose at apical end. ***Mandible*** (Fig. 2) incisors dentate, left with five teeth, right with nine teeth; left ***lacina mobilis*** with eight teeth and eight toothed accessory robust setae, right lacinia mobilis with seven teeth and three accessory robust setae; molar very weakly triturative; palp one-articulate, short and tipped with one slender plumose seta apically. ***Lower lip*** (Fig. 2) inner lobes missing from dissected part, this is uncertain whether they are genuinely lacking or missing during dissection as the animal was very small, outer lobes setose at apical margins. ***Maxilla 1*** (Fig. 2) inner plate small, reaching about two-thirds the length of the outer plate, tipped with two slender, plumose setae; outer plate with seven dentate spine teeth distally; palp two-articulate with three long plumose setae apically. ***Maxilla 2*** (Fig. 2) inner plate shorter and slightly more slender than outer plate, tipped with five long slender, plumose setae, outer plate angled medially and lined with eight long slender plumose setae. ***Maxilliped*** (Fig. 2 – twisted in illustration) inner plate small, with three robust and three slender setae apically; outer plate ovoid, lined with six robust setae medially; palp second article longest, as long as third and fourth articles combined, unguis of dactyl as long as basal part.

**Pereon: *gnathopod 1*** (Fig. 3) subchelate; shorter than gnathopod 1, coxa triangular and longer than pereonite 1, longer than wide, extended ventrally, tapering, acute distally, slightly curved anteriorly, lined with both peg-like projections/setae and long slender, plumose setae, a small additional posterior projection; ratio of article length from basis to dactylus 1.0: 0.1: 0.2: 0.8: 0.5: 0.4; articles ranging from basis to propodus are lined with long slender plumose setae. Basis slender, elongate, margins subparallel, length equal to carpus and merus lengths combined; carpus long, widened distally, setose on both margins; propodus swollen and ovoid, strongly setose, more rounded than proximal articles and produced posteriorly to form a slight rounded heel, palm highly angled not defined but lined with robust setae, palmar margin marked by three robust spines. ***Gnathopod 2*** (Fig. 3) subchelate, longer than gnathopod 1, coxa triangular and longer than pereonite 2, longer than wide, extended ventrally, tapering, acute distally, not curved anteriorly, lined with both peg-like projections/setae and long slender, plumose setae, and robust setae, a reduced additional posterior projection; ratio of article length from basis to dactylus 1.0: 0.1: 0.4: 0.8: 1.0: 1.0; articles ranging from basis to propodus with sparse long slender plumose setae, propodus more rounded than proximal articles and produced posteriorly to form a slight heel, palm highly angled not defined but with only sparse robust and plumose setae. ***Pereopod 3*** (Figs 1, 3) margins of all articles sparsely lined with long slender plumose setae; coxa small and triangular, tipped with two long plumose slender setae at apex, ratio of length of coxa to dactylus 1.0: 4.6: 0.6: 1.5: 4.4: 2.2: 1.5; dactylus tipped with a bifid unguis that is 0.2× length of whole dactylus. ***Pereopod 4*** (Figs 1, 3) margins sparsely lined with long slender plumose setae; coxa small and lobate, giving the impression of being triangular, tipped with three long plumose slender setae at apex, ratio of length of coxa to dactylus 1.0: 3.8: 0.6: 1.0: 3.1: 2.4: 2.8, dactylus tipped with a bifid unguis that is 0.1× length of whole dactylus. ***Pereopod 5*** (Figs 1, 3) margins sparsely lined with long slender plumose setae and occasional peg-like setae; coxa small and triangular, tipped with four long plumose slender setae at apex, ratio of length of coxa to carpus 1.0: 3.4: 0.5: 0.9: 2.7, propodus and dactylus broken off. ***Pereopod 6*** (Figs 1, 3) damaged, broken between ischium and merus, margins sparsely lined with long slender plumose setae and sparse peg-like setae; coxa small and triangular, tipped with one long plumose slender seta at apex, ratio of length of coxa to dactylus 1.0: 4.2: broken: broken: 5.1: 3.1: 2.5, dactylus tipped with bifid unguis 0.1 × of whole dactylus. ***Pereopod 7*** (Figs 1, 3) coxa small and rounded, tipped with one long slender plumose seta, distal articles broken off at carpus.

**Urosome** (Figs 1, 4): uropods slender and elongated, biramous, rami styloid. ***Uropod 1*** (Figs 1, 4) longest of the three uropods, twice the length of uropod 3, peduncle 0.4× as long as the shorter outer ramus, strong peg-like robust setae lining the peduncle and rami, outer ramus slightly shorter than inner ramus. ***Uropod 2*** (Figs 1, 4) peduncle 0.3× length of shorter outer ramus; outer ramus slightly shorter, 0.8× inner ramus; both rami with spines on margins and apical tips. ***Uropod 3*** (Figs 1, 4) peduncle very short, 0.3× shorter than inner ramus, outer ramus slightly shorter than inner, with robust setae; rami slightly damaged at tip. ***Telson*** (Fig. 4) slightly longer than broad; cleft 60% of length, widely cleft u-shaped; apices each bearing three long slender plumose setae.

##### Remarks.

*Lepechinelloides polymetallica* sp. nov. fits in the genus due to the diagnostic characters of the genus. These include large processes located on the dorsal midline, head lacking eyes, rostrum and cephalic teeth, urosome segments 2 and 3 fused, mandibular palp one-articulate and the uropod rami immensely long. It differs from the genus diagnosis by the proportion of antenna 1 peduncle article 2 to article 3, which is subequal in the original diagnosis and distinctly twice as long in this new species. It has been decided by the authors to place this new species in the genus *Lepechinelloides* despite the differences in the diagnosis. This decision is mainly due to the fact this is a single specimen of only 3 mm in length. Until more material is available this species should be placed in *Lepechinelloides*. *Lepechinelloides polymetallica* differs from both *L. karii* and *L. weddellensis* by the possession of small upright pointing extensions and reduced setation on all pereonites (versus strong setae and spines and no extensions on the pereonites of *L karii* and *L. weddellensis*), and the reduced dorsal, vertical projection on pleonite 3 (large and posteriorly directed in both *L. karii* and *L. weddellensis*), ventrally directed midventral extensions on pleonites 1–3 (ventrally directed in *L. weddellensis* but angled in *L. karii*), the widened propodi of both gnathopods (narrow and not expanded in both *L. karii* and *L. weddellensis*), the lack of spines on coxa 5–7 and the large perpendicularly extended gland cone on antenna 2.

Despite being both molecularly and morphologically characterised there would be a great benefit having comprehensive molecular information on the other species in this genus. Due to the enigmatic nature of these organisms, this may be relatively difficult. Adding molecular information will eventually allow a deeper understanding of the evolution within the family and the relationships between this family and other amphipods (Lörz et al. 2020).

##### Distribution.

Abyssal Pacific Ocean, Clarion-Clipperton Zone, 4223 m.

##### Molecular data.

COI sequence data for the holotype of *Lepechinelloides polymetallica* sp. nov. is deposited in GenBank under accession number PQ734637. The species has also received a Barcode Index Number from Barcode of Life Data Systems: BOLD:AEB1214(https://doi.org/10.5883/BOLD:AEB1214).

### Key to the *Lepechinelloides* species of the world

**Table:** 

1	Dorsal extension on pereonite 3 directed posteriorly, reaching past the length of urosomite 1	**2**
–	Dorsal extension on pereonite 3 vertical, one-third the length of urosomite 1	***L. polymetallica* sp. nov**.
2	Pereonites covered in dense setae, dorsal projections on all pereonites directed strongly posteriorly	***L. weddellensis* Andres & Brandt, 2001**
–	Pereonites weakly covered in slender setae, dorsal projections on all pereonites directed vertically	***L. karii* Thurston, 1980**

## Supplementary Material

XML Treatment for Lepechinelloides

XML Treatment for Lepechinelloides polymetallica

## Additional information

### Use of AI

There was no use of AI in the production/creation of this paper.

### Author ORCIDs

Rachael A. Peart https://orcid.org/0000-0001-5968-0811

Anne-Nina Lörz https://orcid.org/0000-0001-9942-349X

### Data availability

All of the data that support the findings of this study are available in the main text.
